# Supplementary material for: The secretome of the environmental bacterium Pseudomonas protegens PBL3 has broad-spectrum antimicrobial activity against plant and human pathogenic bacteria
Source: Microbiol Spectr. 2025 Nov 26;14(1):e02669-25. doi: 10.1128/spectrum.02669-25 (PMC12772282; doi:10.1128/spectrum.02669-25)
Supplement: Supplemental material — Tables S1 and S2; Fig. S1 and S2. [file spectrum.02669-25-s0001.pdf]

Supplementary Table 1: Average growth of plant pathogenic bacteria in the presence of the *P. protegens* PBL3 secretome

Bacterial strains	Control (OD_{600}) (Mean ± SD)	*P. protegens* PBL3 Secretome (OD_{600}) (Mean ± SD)
B. glumae UAPB13	1.66 ± 0.15	0.12 ± 0.01
B. glumae UAPB10	2.05 ± 0.08	0.22 ± 0.05
B. glumae UAPB11	1.57 ± 0.09	0.35 ± 0.03
B. gladioli CU3082	1.06 ± 0.01	0.57 ± 0.04
B. gladioli CU3083	1.58 ± 0.55	0.72 ± 0.18
B. gladioli CU3891	2.39 ± 0.08	0.86 ± 0.03
B. cenocepacia CU0318	2.39 ± 0.14	0.59 ± 0.03
B. cenocepacia CU3094	2.49 ± 0.09	1.71 ± 0.27
B. cenocepacia CU3368	2.94 ± 0.05	1.23 ± 0.09
B. cenocepacia CU3370	2.91 ± 0.10	0.80 ± 0.11
B. cenocepacia CU6878	2.95 ± 0.20	0.97 ± 0.06
B. cenocepacia CU3371-1	2.29 ± 0.18	0.95 ± 0.11
B. cenocepacia CU3371-2	2.96 ± 0.57	0.46 ± 0.02
B. sp. O64a	2.01 ± 0.24	0.32 ± 0.03
X. campestris pv. *malvacearum*	1.08 ± 0.17	1.10 ± 0.22
E. amylovora	1.58 ± 0.10	1.80 ± 0.00
X. axonopodis	2.23 ± 0.13	2.52 ± 0.06

Supplementary Table 2: Average growth of human pathogenic bacteria in the presence of the *P. protegens* PBL3 secretome

Bacterial strains	Phosphate-buffered saline (Mean ± SD)			*P. protegens PBL3* secretome (Mean ± SD)		
	**10%(v/v)**	**20 %(v/v)**	**30 %(v/v)**	**10 %(v/v)**	**20 %(v/v)**	**30 %(v/v)**
A. baumanii	0.52 ± 0.06	0.14 ± 0.05	0.06 ± 0.03	0.34 ± 0.14	0.01 ± 0.00	0.002 ± 0.00
E. coli O157:H7	1.11 ± 0.02	1.04 ± 0.03	0.91 ± 0.04	0.85 ± 0.08	0.62 ± 0.03	0.56 ± 0.06
P. aeruginosa PA01	1.02 ± 0.08	1.13 ± 0.25	0.94 ± 0.20	1.15 ± 0.09	1.12 ± 0.25	0.47 ± 0.04
P. aeruginosa PA103	0.91 ± 0.23	0.91 ± 0.11	0.48 ± 0.00	0.88 ± 0.06	0.85 ± 0.06	0.38 ± 0.02
S. typhi	1.07 ± 0.03	1.03 ± 0.01	0.98 ± 0.01	1.11 ± 0.05	0.96 ± 0.01	0.93 ± 0.08
Y. enterocolitica	0.45 ± 0.04	0.45 ± 0.10	0.44 ± 0.03	0.44 ± 0.08	0.02 ± 0.00	0.02 ± 0.00
E. faecium	1.11 ± 0.03	1.04 ± 0.01	1.15 ± 0.03	0.78 ± 0.04	0.79 ± 0.04	0.51 ± 0.03
L. innocua	1.03 ± 0.25	1.11 ± 0.18	0.99 ± 0.39	1.09 ± 0.41	0.83 ± 0.31	0.66 ± 0.33
S. aureus	1.64 ± 0.21	1.63 ± 0.14	1.57 ± 0.06	1.68 ± 0.07	1.14 ± 0.15	1.10 ± 0.22
E. coli K12	0.45 ± 0.12	0.35 ± 0.08	0.29 ± 0.10	0.45 ± 0.05	0.34 ± 0.09	0.33 ± 0.28

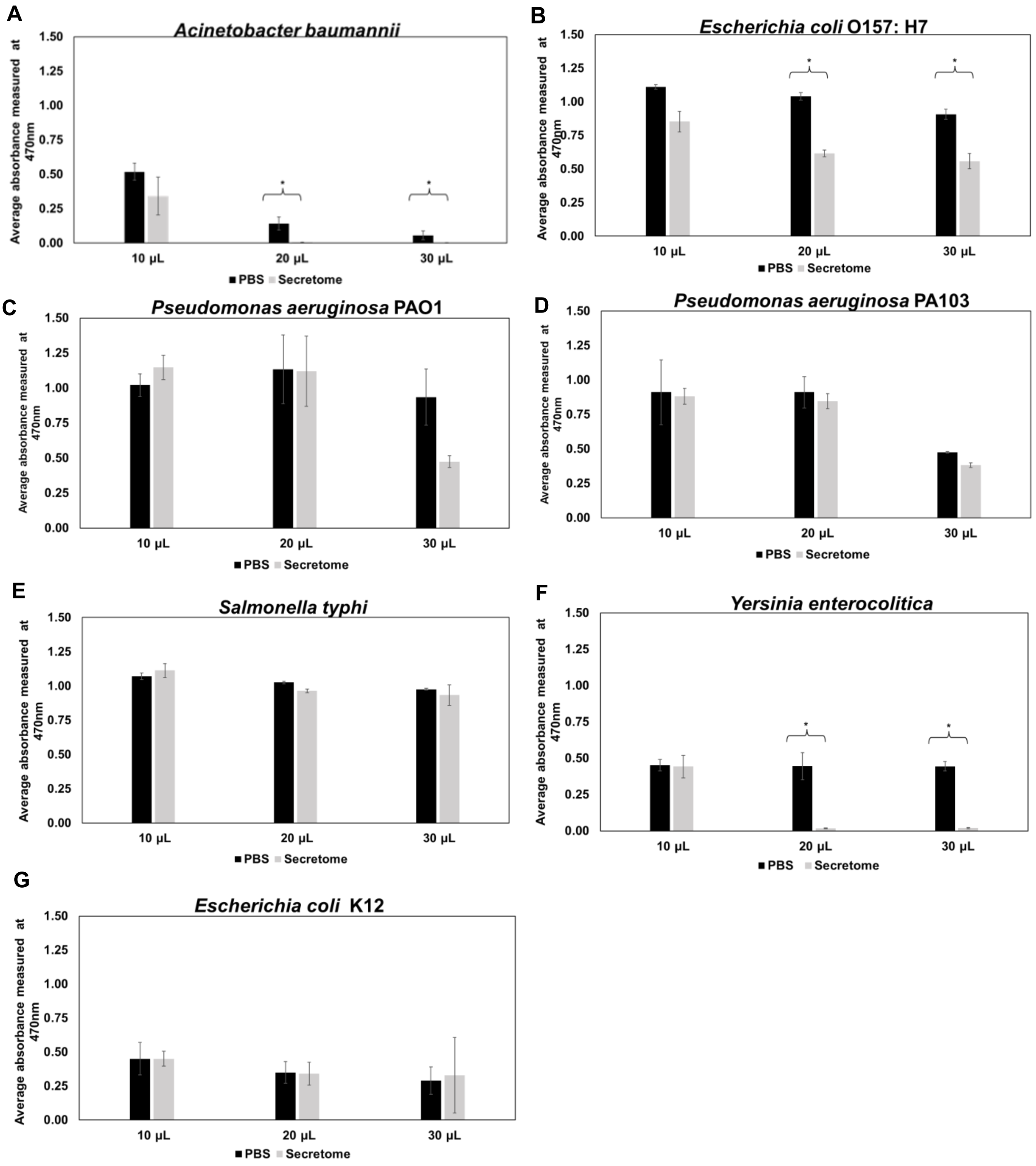

Supplementary Figure 1. Screening the antimicrobial property of *P. protegens* PBL3 secretome against gram-negative human pathogens. Pathogenic gram-negative bacteria were grown in agar plates for 20 hours. Bacteria was scraped from a plate and diluted in Phosphate-buffered saline (PBS) buffer to an optical density 600 (OD_{600}) of 0.2 and further diluted in growing media to a final concentration of 1.5 X 10^6 CFU/mL. Different volumes of the *P. protegens* PBL3 secretome or PBS were mixed with different volumes of growing media to a final volume of 100 µl and grown for 18h at 37oC with constant agitation. After 18 h, 0.1% 2,3,5-Triphenyl-tetrazolium chloride was added to the cultures. Absorbance at 470 nm was read in a plate reader. Bar represents the average absorbance of bacteria minus the absorbance of media alone, and the error bar represents the standard deviation of six replicates. Asterisk suggests a difference between the PBS and PBL3 secretome, with a level of significance set at p=0.05.

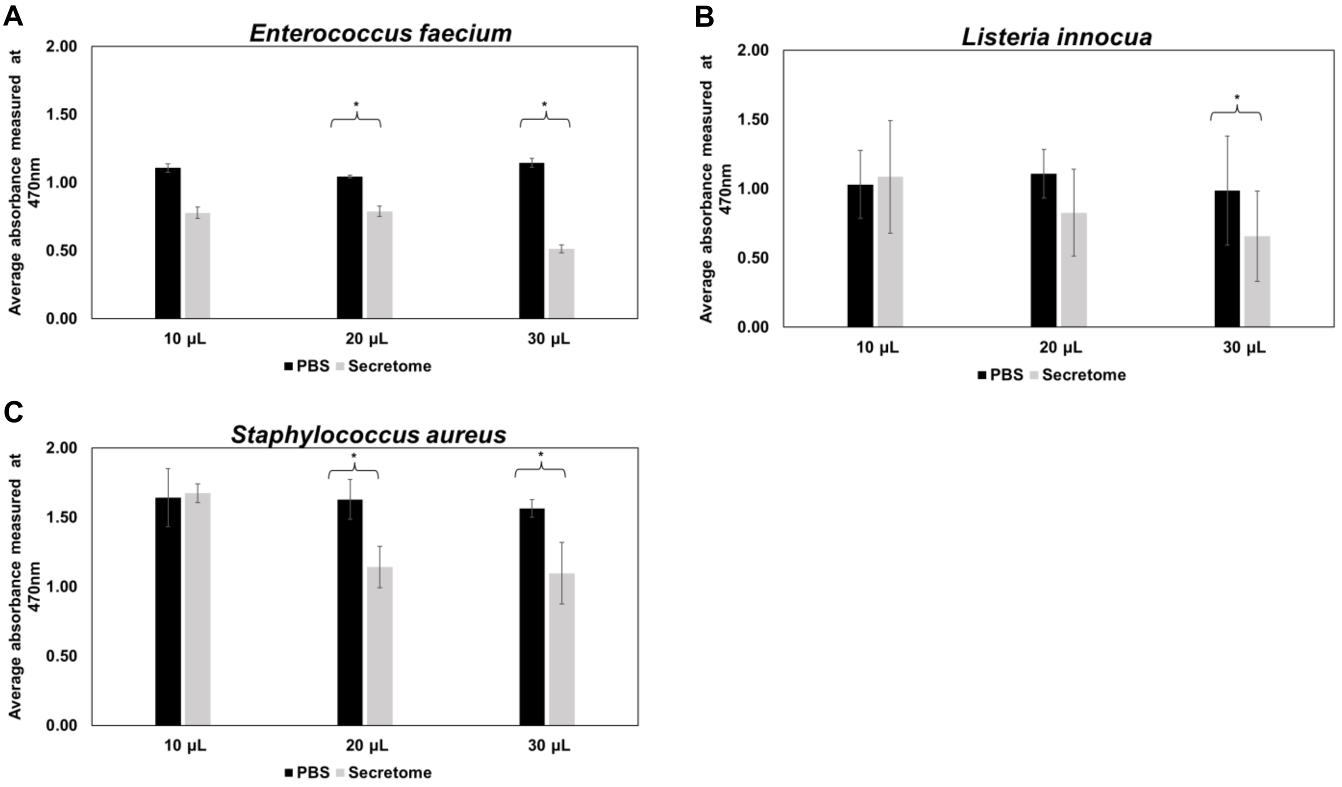

Supplementary Figure 2. Screening the antimicrobial property of *P. protegens* PBL3 secretome against gram-positive human pathogens. Pathogenic gram-positive bacteria were grown in agar plates for 20 hours. Bacteria were scraped from a plate and diluted in Phosphate-buffered saline (PBS) buffer to an optical density 600 (OD_{600}) of 0.2 and further diluted in growing media to a final concentration of 1.5 X 10^6 CFU/mL. Different volumes of the *P. protegens* PBL3 secretome or PBS were mixed with different volumes of growing media to a final volume of 100 µl and grown for 18h at 37°C with constant agitation. After 18 h, 0.1% 2,3,5-Triphenyl-tetrazolium chloride was added to the cultures. Absorbance at 470 nm was read in a plate reader. Bar represents the average absorbance of bacteria minus the absorbance of media alone, and the error bar represents the standard deviation of six replicates. Asterisk suggests a difference between the PBS and PBL3 secretome with a level of significance set at p=0.05.
